# Riboswitch RS*_thiT_* as a Molecular Tool in Lactococcus lactis

**DOI:** 10.1128/aem.01764-21

**Published:** 2022-02-22

**Authors:** Eduardo P. Hernández-Ortega, Sjoerd van der Meulen, Lucas J. Kuijpers, Jan Kok

**Affiliations:** a Department of Molecular Genetics, Groningen Biomolecular Sciences and Biotechnology Institute, University of Groningen, Groningen, The Netherlands; University of Helsinki

**Keywords:** riboswitch, TPP, thiamine, *L. lactis*, RNA-based device, molecular tool

## Abstract

Previous RNA sequencing has allowed the identification of 129 long 5′ untranslated regions (UTRs) in the Lactococcus lactis MG1363 transcriptome. These sequences potentially harbor *cis*-acting riboswitches. One of the identified extended 5′ UTRs is a putative thiamine pyrophosphate (TPP) riboswitch. It is located immediately upstream of the thiamine transporter gene *thiT* (*llmg_0334*). To confirm this assumption, the 5′-UTR sequence was placed upstream of the gene encoding the superfolder green fluorescent protein (sfGFP), *sfgfp*, allowing the examination of the expression of sfGFP in the presence or absence of thiamine in the medium. The results show that this sequence indeed represents a thiamine-responsive TPP riboswitch. This RNA-based genetic control device was used to successfully restore the mutant phenotype of an L. lactis strain lacking the major autolysin gene, *acmA*. The L. lactis *thiT* TPP riboswitch (RS*_thiT_*) is a useful molecular genetic tool enabling the gradual downregulation of the expression of genes under its control by adjusting the thiamine concentration.

**IMPORTANCE** The capacity of microbes with biotechnological importance to adapt to and survive under quickly changing industrial conditions depends on their ability to adequately control gene expression. Riboswitches are important RNA-based elements involved in rapid and precise gene regulation. Here, we present the identification of a natural thiamine-responsive riboswitch of Lactococcus lactis, a bacterium used worldwide in the production of dairy products. We used it to restore a genetic defect in an L. lactis mutant and show that it is a valuable addition to the ever-expanding L. lactis genetic toolbox.

## INTRODUCTION

Lactococcus lactis is a member of the large group of industrially relevant lactic acid bacteria (LAB). It has already been employed for millennia in the fabrication of fermented milk products, where it serves a role in producing flavor compounds and lactic acid. As such, it plays an important role in the organoleptic quality and preservability of dairy products. Due to its important function in milk fermentation, L. lactis has become the model for LAB. It quickly gained this status once genetic engineering technology was established in this organism and because it has a small genome (2.3 Mbp), is easy to work with in the laboratory, and is generally recognized as safe (GRAS). Molecular tools such as host cloning and inducible gene expression systems as well as efficient gene knockout, knock-in, and knockdown strategies are readily available (1–4). The further development of molecular tools should allow the exploration and exploitation of the capabilities of this organism even further, in biotechnology and as a microbial cell factory.

DNA and DNA-binding proteins are the most common devices that molecular biologists use to control gene expression in cells. Although RNA-based tools have reasonably lagged for this purpose, the recognition that RNA can also play an important regulatory role, at both the transcriptional and the posttranscriptional levels, has led to an intensification of research on regulatory systems based on RNA molecules (5). One case is that of the RNA-based genetic switches, or riboswitches, which use an input signal to regulate gene expression. The signals are small molecules such as amino acids, vitamins, nucleotides, or metal ions. Their interaction with their cognate riboswitch can modify gene expression at both the transcriptional and translational levels (6).

Riboswitches possess two distinguishing domains: the aptamer domain, which senses the ligand, is a highly folded structure that classifies the riboswitch, while the expression platform (also called the actuator domain) is the site of the conformational change that modifies gene expression (7). Genes controlled by riboswitches frequently encode proteins involved in the biosynthesis or transport of the molecule being sensed (8). The versatile cellular tasks of RNA molecules have permitted the engineering of natural RNA-based devices into synthetic devices, allowing programmable genetic responses directly from metabolites without the assistance of proteins. This represents an important advantage of riboswitch-based molecular tools (9). To date, around 40 different classes of riboswitches have been experimentally validated in several bacterial species (10).

Previous work on the investigation of the transcriptomic landscape of L. lactis by RNA sequencing has uncovered the existence of 129 long 5′ untranslated regions (UTRs) carrying potential regulatory elements (11). Putative riboswitches for purine, pyrimidine, lysine, fluoride, flavin mononucleotide, and thiamine pyrophosphate (TPP) were detected. TPP-binding riboswitches have been found in the 5′ UTRs of genes involved in thiamine biosynthesis; they respond with high affinity to TPP (12). TPP riboswitches operate by two distinct gene regulation mechanisms, namely, transcription termination and the prevention of translation (13).

In L. lactis, three long 5′ UTRs upstream of genes involved in thiamine metabolism were detected. We selected the putative riboswitch of the gene *thiT*, a characterized gene in L. lactis. It encodes the 22.7-kDa high-affinity S-component thiamine transporter (14). To examine whether the 5′ UTR of *thiT* represents a genuine TPP riboswitch, we placed it upstream of the superfolder green fluorescent protein (sfGFP) reporter gene *sfgfp* in an integration plasmid and inserted the construct into the chromosome of L. lactis to characterize it. We also explored the possibility of using it as a molecular tool in L. lactis, by regulating the expression of an endogenous gene.

## RESULTS

### Three possible TPP riboswitches in L. lactis.

**FIG 1:**
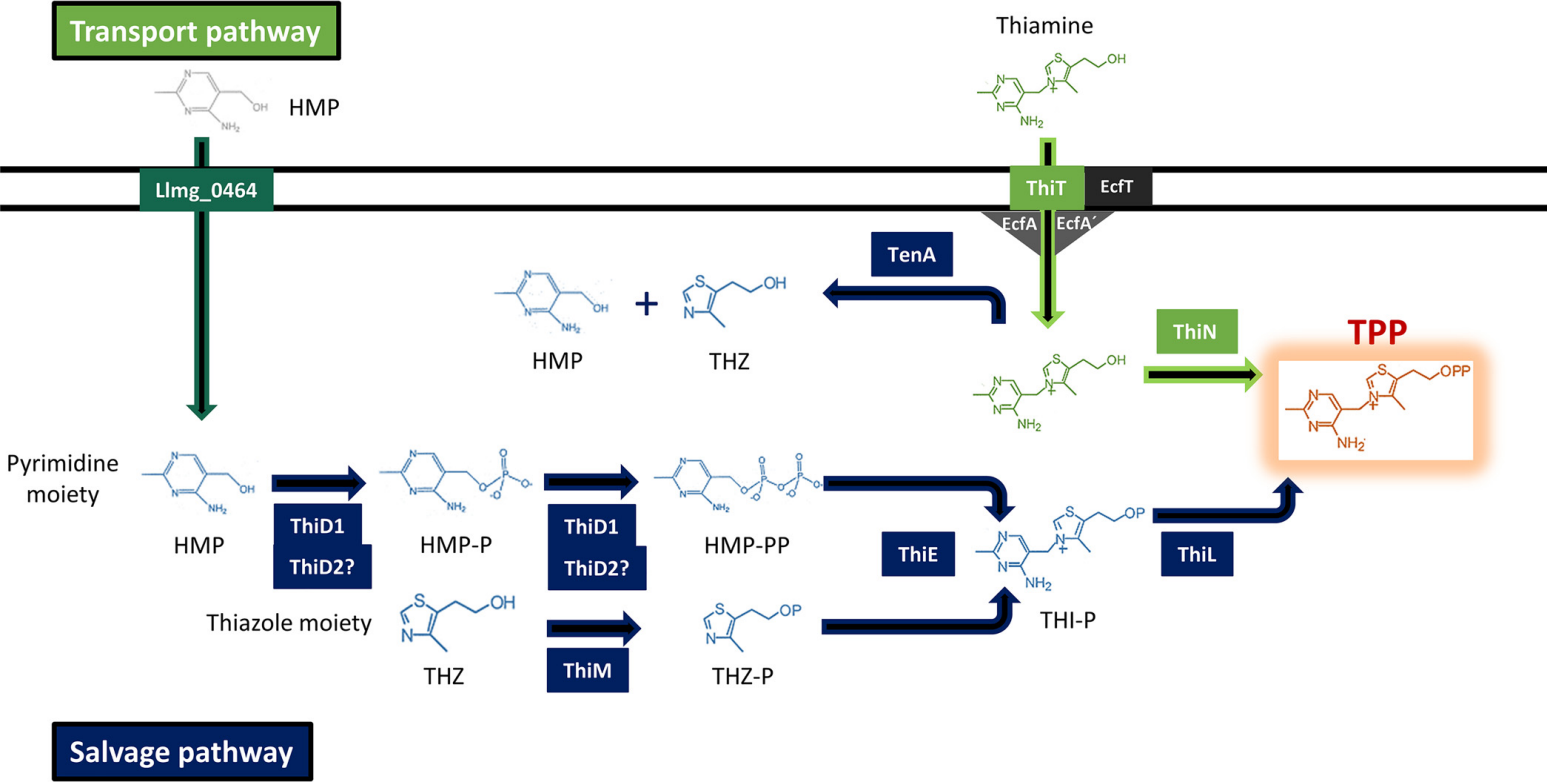
Putative pathways of thiamine in L. lactis. In green is the transport pathway. Thiamine or precursors are taken up by the high-affinity core transporter ThiT. Thiamine is converted to thiamine pyrophosphate (TPP) by ThiN, the thiamine pyrophosphokinase. The putative *llmg_0464* product could be involved in hydroxymethyl pyrimidine (HMP) uptake (55). In blue is the salvage pathway. Thiazole alcohol (THZ) and HMP are phosphorylated by ThiM and ThiD1, respectively. THZ and HMP can also be obtained from thiamine through hydrolysis by TenA. After a number of phosphorylation steps performed by ThiM and ThiD1 (and possibly ThiD2), the products are coupled to thiamine monophosphate (Thi-P) by ThiE. Finally, Thi-P is phosphorylated by ThiL to obtain TPP. EcfA and EcfA′ are the homologous ATPases, and EcfT is a transmembrane protein. Together with the S components ThiT and Llmg_0464, they form a group II ECF transporter (15). Abbreviations: THZ-P, thiazole phosphate; HMP-P, hydroxymethyl pyrimidine phosphate; HMP-PP, hydroxymethyl pyrimidine pyrophosphate.

Previously, we identified by differential RNA sequencing three long 5′ UTRs upstream of genes/operons putatively involved in thiamine metabolism in L. lactis MG1363 (11). Two of these may contain a TPP riboswitch, one in the 158-nucleotide (nt) 5′ UTR upstream of the gene *thiT* (*llmg_0334*) and one in the 5′ UTR of 196 nt upstream of the operon *thiMD1E* (*llmg_1216–18*). The *thiI* gene (*llmg_0395*) carries a 5′ UTR of 122 nt. Considering the role of their homologs in Escherichia coli and Bacillus subtilis (14), all these genes are predicted to play a role in the biosynthesis of TPP in L. lactis. The possible pathway for TPP production in L. lactis is shown in Fig. 1. Little is known about thiamine biosynthesis in L. lactis. Of the genes mentioned above, only the product of the *thiT* gene has been studied in detail. ThiT is a high-affinity thiamine transporter (15).

**FIG 2:**
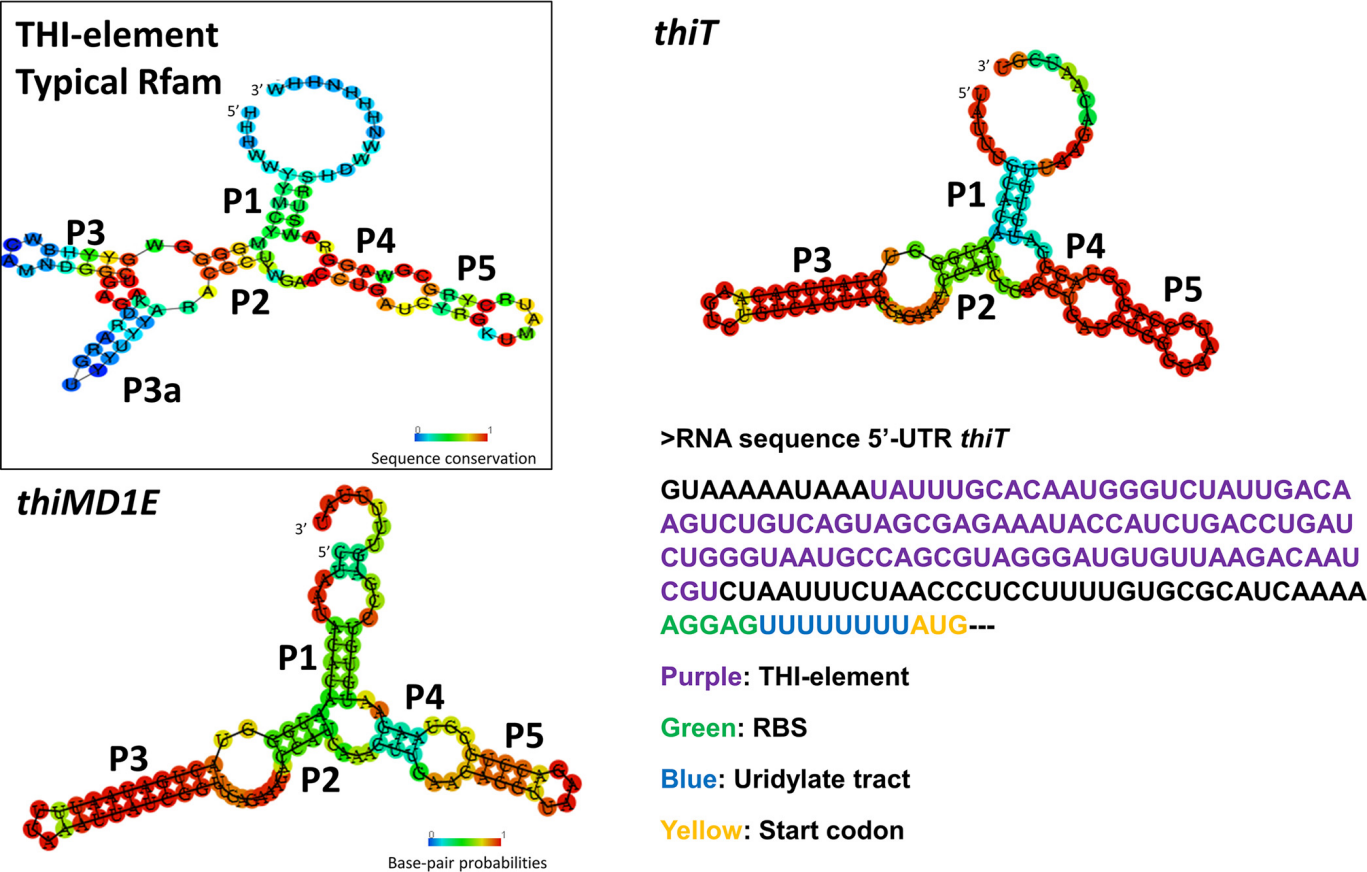
Secondary structure predictions of the THI element, with start/end positions at nt 12/111 of L. lactis *thiT* (*llmg_0334*) and nt 17/117 of the L. lactis operon *thiMD1E* (*llmg_1216*), as predicted by RNAfold by base-pair probabilities (18) (http://rna.tbi.univie.ac.at). In the top left corner, the conventional TPP riboswitch structure (17) is shown with its five stem-loops (P1 to P5). The complete sequence of the 5′ UTR of *thiT* is shown.

TPP riboswitches possess highly conserved sequences and structures (16). The Rfam website was used to confirm, by bit score, the presence of so-called THI (thiamine-regulatory) elements (17). They are present in the upstream regions of *thiT* and *thiMD1E* (for the THI element in the 5′ UTR at start/end positions 12/111, bit score of 62.4 and E value of 4.8e−12; for the THI element at start/end positions 17/117, bit score of 59.1 and E value of 3.5e−11). No clear THI element could be discerned in the 5′ UTR of *thiI* (data not shown). The RNAfold Web server was used to predict the structures of the two THI elements (Fig. 2). Using free-energy minimization for base-pair probabilities, the normalized energies were determined to be −28.3 kcal/mol for the 5′-UTR structure of *thiT* and −23.8 kcal/mol for that upstream of *thiM* (18). The results show that the sequence upstream of L. lactis *thiT* and *thiMD1E* contains the 5 stem-loops (P1 to -5) conserved in THI elements. P1 close to the 5′ side links the aptamer domain to the expression platform, and P2 and P3 interact with the pyrimidine ring in TPP. P3 is the most variable among the species; bacteria and archaea commonly have a P3a stem. A P3a structure is not obvious in the L. lactis THI elements. P4 and P5 bind to the pyrophosphate group of TPP (16, 19).

### The 5′ UTR of L. lactis *thiT* contains a TPP riboswitch.

**FIG 3:**
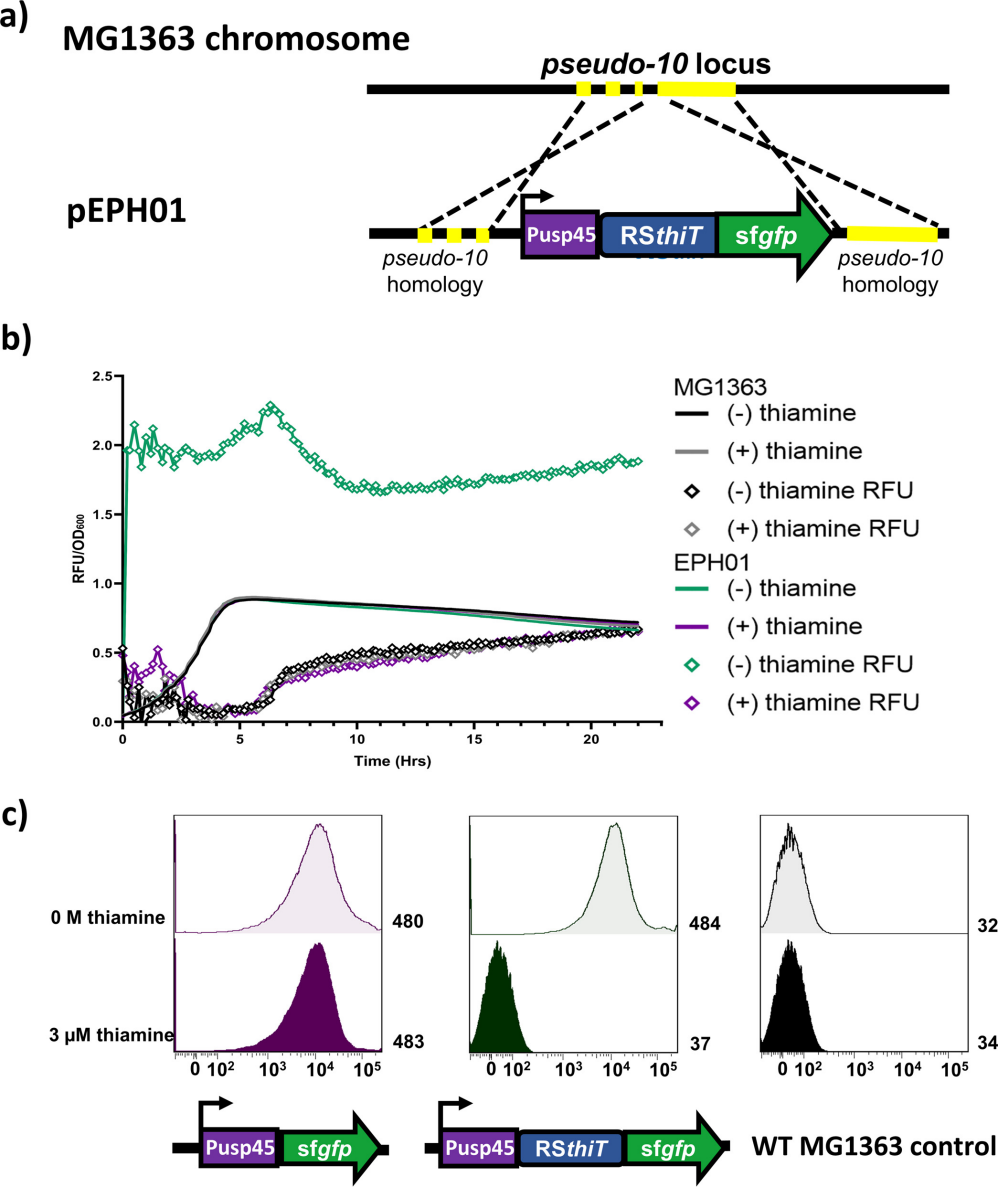
(a) Schematic overview of the integration strategy used to obtain L. lactis EPH01. The relevant part of the pSEUDO_10-Pusp45::RS*_thiT_*::*sfgfp* plasmid and the *pseudo_10* locus of L. lactis MG1363 are shown. Pusp45, promoter of the L. lactis *usp45* gene; RS*_thiT_*, TPP riboswitch in the 5′ UTR of the *thiT* gene; *sfgfp*, superfolder green fluorescent protein gene. (b) Growth (solid lines) and RFU development (diamond lines) in microtiter plate cultures of L. lactis strains EPH01 and MG1363 growing at 30°C in G-CDMPC without (−) and with (+) 3 μM thiamine. Fluorescence was measured in triplicate, and the data are shown as the means ± standard deviations (SD). (c) sfGFP expression, measured by FACS analysis, as a function of the thiamine concentration, in the presence or absence of the 5′ UTR of the gene *thiT*. Ungated events (100,000) for each sample are shown; the mean was used to obtain arbitrary units. WT, wild type.

To analyze the complete sequence of the 5′ UTR of *thiT* (Fig. 2), we used the nonreplicative integration vector pSEUDO_10, a plasmid carrying a strong constitutive L. lactis promoter (Pusp45) upstream of the reporter gene *sfgfp* (20). The entire 158-nt sequence with the putative riboswitch (RS*_thiT_*) was inserted immediately upstream of the AUG start codon of *sfgfp*, resulting in plasmid pSEUDO_10-RS*_thiT_*::*sfgfp* (Fig. 3a). This plasmid was used to insert the genetic construct, via double-crossover integration, into the transcriptionally silent *pseudo_10* locus in the chromosome of L. lactis MG1363. The L. lactis strain EPH01 obtained in this way was grown in a chemically defined medium without or with 3 μM thiamine. As is clear (Fig. 3b), the cells produce sfGFP activity in the absence of thiamine. The presence of RS*_thiT_ per se* does not influence the expression of sfGFP (Fig. 3c). When thiamine is present, the fluorescence signal is in the same range as the background level in the parent strain L. lactis MG1363. These results indicate that 5′-UTR-labeled RS*_thiT_* of the gene *thiT* is indeed a TPP riboswitch with which gene expression can be easily switched on or off.

### RS*_thiT_* shows a graded response to the thiamine concentration.

**FIG 4:**
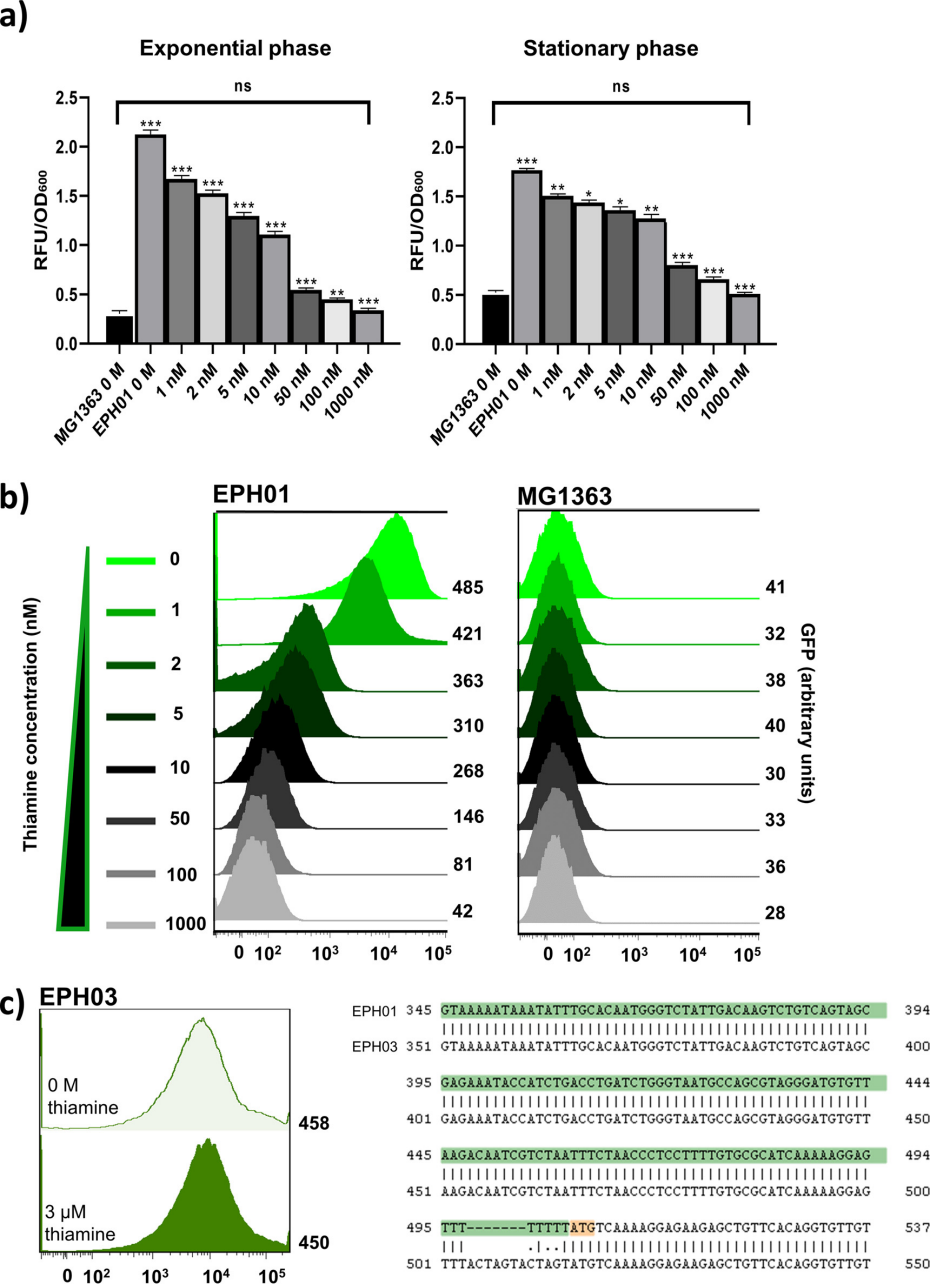
RS*_thiT_* riboswitch activity as a function of the thiamine concentration. (a) Spectrophotometry analysis of exponential (OD_600_ of 0.4)- and stationary (OD_600_ 0.8)-phase cultures of L. lactis EPH01. RFU, relative fluorescence units. Bars represent mean values, with standard errors indicated. Each bar shows a statistically significant value compared to the previous concentration, measured independently by multiple-comparison ANOVA (*, *P* < 0.05; **, *P* < 0.001; ***, *P* < 0.0001; ns, not significant). All bars are the means from three independent experiments, each consisting of three technical replicates. (b) Single-cell fluorescence flow cytometry (by FACS) analysis. Strains MG1363 and EPH01 were grown in G-CDMPC with different concentrations of thiamine. (c) Analysis of the U-tract mutant L. lactis EPH03. (Left) FACS analysis of sfGFP expression as a function of the thiamine concentration. (Right) Mutation in EPH03 in comparison to the sequence in L. lactis EPH01. For panels b and c, fluorescence measurements were taken at exponential phase (OD_600_ = 0.4). Ungated events (100,000) for each sample are shown; the mean was used to obtain arbitrary units.

To investigate the responsiveness of RS*_thiT_*, sfGFP expression in L. lactis EPH01 was analyzed at various concentrations of thiamine in the medium. The sfGFP signal was measured *in vivo* in exponential-phase cultures (Fig. 4a). No significant statistical difference was observed at 1 μM thiamine between the control strain MG1363 and L. lactis EPH01. This concentration was required to decrease the sfGFP fluorescence to the background level of L. lactis MG1363. At 1 nM thiamine, the first reduction of the sfGFP signal was observable. A graded response to the thiamine concentration was seen between 1 nM and 1 μM (Fig. 4a). Similar results were obtained with stationary-phase cultures, albeit the sfGFP levels were generally lower, and the response was less well graded. This is most probably due to the accumulation and stability of the sfGFP protein (Fig. 4a). In conclusion, *RS_thiT_* allows the regulation of gene expression in L. lactis by adjusting the extracellular concentration of thiamine.

Next, we examined sfGFP expression under the control of RS*_thiT_* in exponential-phase single cells. To obtain a quantitative range of action of RS*_thiT_*, 1 × 10^5^ cells per sample were examined by flow cytometry (Fig. 4b). The analysis showed an ∼10-fold range of action between the cells grown without thiamine and the controls: cells of strain MG1363 or strain EPH01 in the presence of 1 μM thiamine. In accordance with the data on the culture level (Fig. 4a), the flow cytometry data show a gradual response to the gradient of thiamine (Fig. 4b).

As can be seen in Fig. 2, a stretch of uridine residues immediately upstream of the AUG start codon could constitute an intrinsic Rho-independent transcriptional terminator. A mutation in this region, in which part of the U stretch was replaced by an SpeI recognition sequence, resulted in the constitutive expression of sfGFP, independent of the thiamine concentration (Fig. 4c). The importance of this sequence for the function of TPP riboswitches has been reported previously (21). Structural biology studies have demonstrated that riboswitches are tuned across a gradient of activities to control gene expression (22, 23). This possibility, namely, to fine-tune gene expression, should be an advantage in using RS*_thiT_* as a molecular tool.

### L. lactis RS*_thiT_*, a useful molecular tool.

**FIG 5:**
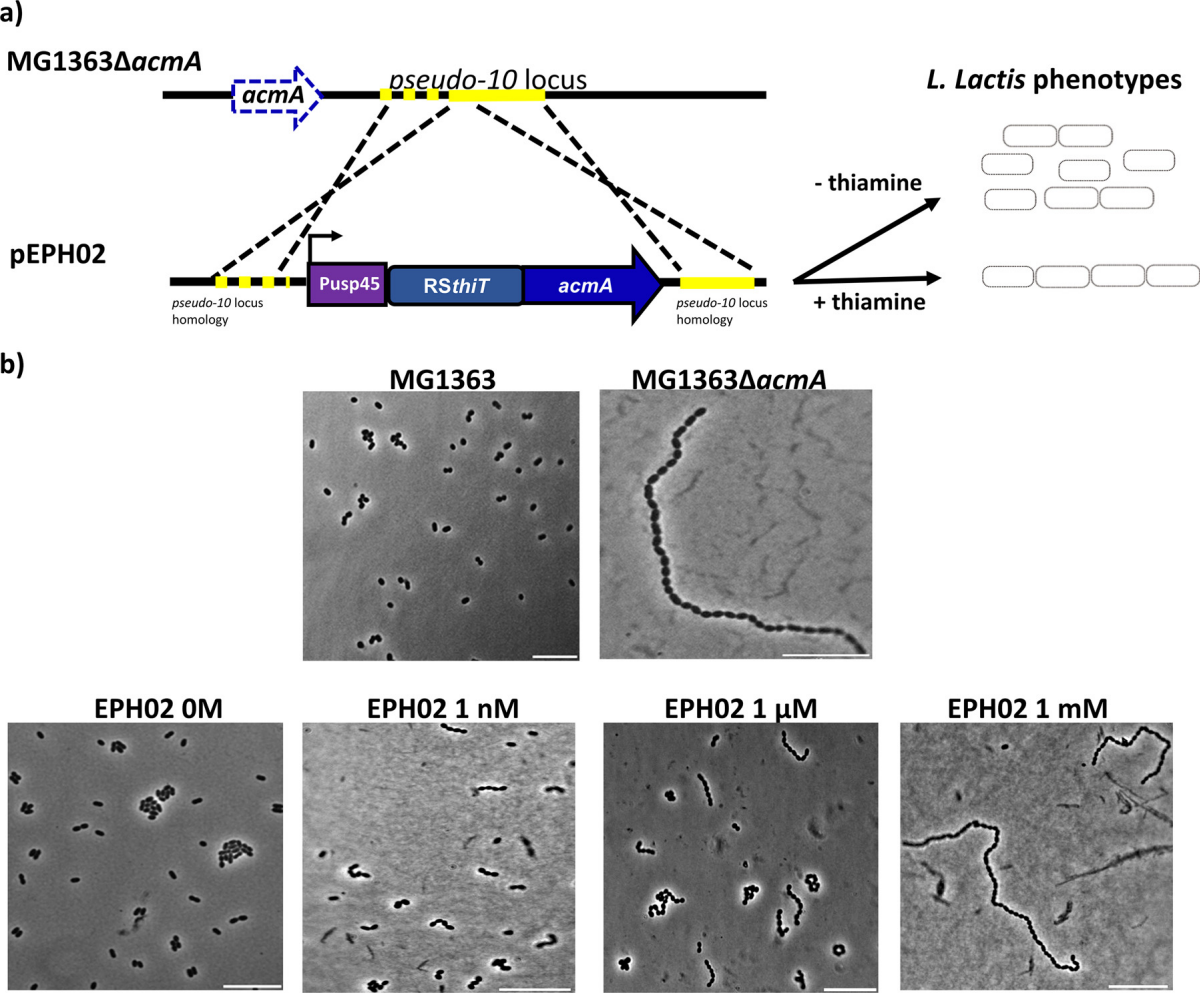
(a) Integration plasmid pSEUDO_10-Pusp45::RS*_thiT_*::*acmA* used to construct L. lactis EPH02 carrying the Pusp45::RS*_thiT_*::*acmA* construct in the *pseudo_10* locus (yellow). Integration was done in L. lactis MG1363 Δ*acmA*, which carries a deletion in the major autolysin gene *acmA* (broken arrow). Pusp45, promoter of the L. lactis *usp45* gene; RS*_thiT_*, TPP riboswitch. (b) Phase-contrast microscopy of samples of the indicated cultures, grown to mid-exponential phase at 30°C in G-CDMPC. The top two panels show cells grown without thiamine; the bottom panels show cells grown in the presence of the indicated concentrations of thiamine. Bars, 10 μm.

To assess the suitability of L. lactis RS*_thiT_* as a genetic control device, it was employed to drive the expression of the endogenous gene *acmA*. This gene encodes the major peptidoglycan hydrolase of L. lactis, an enzyme that is necessary for cell wall synthesis and proper cell separation (24). An L. lactis MG1363 Δ*acmA* mutant forms long chains of cells, a specific phenotype that can be easily monitored. The chromosomal integration vector pSEUDO_10-RS*_thiT_*::*acmA* (pEPH02) was constructed by replacing the *sfgfp* gene in pEPH01 with the coding sequence of *acmA* (Fig. 5a).

Plasmid pEPH02 was used to introduce in L. lactis Δ*acmA*, by double-crossover integration in the *pseudo_10* locus, a single copy of the *acmA* gene with the *RS_thiT_* sequence in its 5′ UTR. The strain obtained in this way was named L. lactis EPH02. As shown previously (25), the Δ*acmA* mutant grows as long chains of cells in the exponential phase of growth, while the parent strain MG1363 is mainly present as duplicate cells (duplos) (Fig. 5b). In a culture of L. lactis EPH02 growing without thiamine, mainly duplos of cells are visible, indicating that the *acmA* gene is transcribed and translated under these circumstances (Fig. 5b). The addition of thiamine to the culture resulted in the formation of chains of cells. By increasing the concentration of thiamine, the mutant long-chain phenotype was gradually recovered; thiamine concentrations of between 1 nM and 1 μM led to an intermediate chaining phenotype. The long-chain phenotype was completely recovered when L. lactis EPH02 was grown with 1 mM thiamine (Fig. 5b). These results show that RS*_thiT_* of L. lactis can be used to gradually downregulate gene expression. It should be valuable as a device in synthetic gene regulation networks and in metabolic engineering strategies in this organism.

## DISCUSSION

Thiamine pyrophosphate is an essential enzyme cofactor in all living systems. Its biosynthesis involves a multistep pathway in most bacteria, yeast, and plants (26). The genes required for the initial steps of a *de novo* pathway are absent from the L. lactis genome (14). The thiamine transporter ThiT of L. lactis is a member of the energy-coupling factor (ECF) subclass of transporters with an unusually high affinity for thiamine (dissociation constant [*K_d_*] = 120 pM) (27, 28). Thiamine pyrophosphate, thiamine monophosphate, and pyrithiamine precursors are all also bound, with nanomolar affinity (15). After thiamine has been taken up, it is transformed by thiamine pyrophosphokinase, the product of *thiN*, to TPP (29).

Of the three 5′ UTRs in genes/operons involved in thiamine biosynthesis in L. lactis, the one upstream of *thiT* is confirmed here to be a TPP riboswitch (RS*_thiT_*). RS*_thiT_* is the fourth confirmed riboswitch in L. lactis and the first to be used as a molecular tool (30–32). Bacterial TPP riboswitches control their downstream genes by either inducing transcription termination, inhibiting translation initiation, or both, while in eukaryotes, they operate by promoting alternative splicing (33, 34). At this stage, it is not known to which of the TPP-related compounds L. lactis RS*_thiT_* responds best. The ligand with the highest affinity *in vitro* for the E. coli and B. subtilis TPP riboswitches is TPP, the biologically active form of thiamine. Some pyrithiamine precursors and thiamine itself can also interact with the aptamer domain (35–38). This versatility of RNA to interact with various ligands with different affinities is a relevant feature to be explored to use the TPP riboswitch as a molecular tool.

Riboswitches are of great interest in the continuous development of biosensors and other innovative molecular tools. Natural as well as synthetic riboswitches have been considered devices that might overcome the limitations of protein-based biosensors (39, 40). RNA-based molecular tools are versatile and combine a fast response with higher specificity and sensitivity than protein-protein interaction biosensors (41, 42). In addition, they can be quickly and flexibly engineered *in vitro*, and combined with other RNA devices such as the various CRISPR-Cas systems, they can even be used to explore novel biological queries. For instance, the expression platform of the E. coli TPP riboswitch was swapped with the Spinach aptamer, allowing TPP binding to induce Spinach fluorescence and the discovery of agonists and antagonists of the riboswitch using a simple fluorescence readout (43).

Novel types of natural and synthetic RNA devices are important to increase the molecular genetic toolbox, e.g., for the expression of (heterologous) lethal proteins, to gradually silence essential genes that cannot be deleted from the chromosome. In the case of the natural L. lactis RNA-based control device RS*_thiT_* presented here, this can be done simply by adjusting the thiamine concentration in the medium. We successfully employed RS*_thiT_* to regulate the expression of two downstream genes, *sfgfp* and *acmA*. For *sfgfp*, 1 μM thiamine in the medium is enough to turn the device off. A higher concentration of 1 mM is needed to switch off *acmA*, which we assume is because AcmA is a very active enzyme. The range of action of RS*_thiT_*, as measured by fluorescence-activated cell sorter (FACS) analysis, is approximately 10-fold, which is higher than those of natural neomycin riboswitches obtained in a mutant screening effort (∼7.5-fold in the presence of neomycin), or ∼4-fold in the riboswitch controlling the thiamine transporter *DUR31* of Candida parapsilosis (44, 45). It is modest in comparison with the robust synthetic theophylline riboswitch (∼96-fold) from E. coli, where the key to these improvements involved optimizing the expression platform, including the ribosome-binding site (RBS) (46). We anticipate that L. lactis RS*_thiT_* will permit the construction of increasingly complex genetic circuits for biotechnology.

## MATERIALS AND METHODS

### Bacterial strains, plasmids, and growth conditions.

Table 1 presents the strains and plasmids employed in this study. E. coli DH5α (Life Technologies, Gaithersburg, MD, USA) was used as a host for the construction and purification of pSEUDO_10-based plasmids. E. coli was grown at 37°C in TY (1% Bacto tryptone, 0.5% Bacto yeast extract, and 1% NaCl) medium or on TY agar (1.5%) plates. L. lactis was grown at 30°C in chemically defined medium without thiamine (CDMPC), a medium devised to decrease the background fluorescence level (47), or in M17 broth (Difco, Becton, Dickinson, Le Pont de Claix, France), both containing 0.5% (wt/vol) glucose (G-CDMPC [chemically defined medium for prolonged cultivation] and GM17, respectively). L. lactis was plated onto GM17 agar (1.5%) plates. For selection, 10 μg/mL or 150 μg/mL of erythromycin was added to the medium for L. lactis or E. coli, respectively.

**TABLE 1:** L. lactis and E. coli strains and plasmids used in this study

Strain or plasmid	Relevant phenotype or genotype^*a*^	Reference
Strains		
MG1363	L. lactis subsp. *cremoris*; plasmid-free derivative of L. lactis NCDO712	53
Δ*acmA*	MG1363 knockout mutant of autolysin gene *acmA*	23
EPH01	MG1363; Pusp45::RS*_thiT_*::*sfgfp* integrated into the *pseudo_10* locus	This work
EPH02	MG1363; Pusp45::RS*_thiT_*::*acmA* integrated into the *pseudo_10* locus	This work
EPH03	MG1363; Pusp45::RS*_thiT/mut-U-tract_*::*sfgfp* integrated into the *pseudo_10* locus	This work
DH5α	E. coli; d*lacZ*ΔM15 Δ(*lacZYA-argF*)*U169 recA1 endA1 hsdR17*(r_K_^−^ m_K_^+^) *supE44 thi-1 gyrA96 relA1*	54

Plasmids		
pSEUDO_10	Em^r^; pCS1966 derivative for integration into the *llmg_pseudo_10* locus	3
pSEUDO_10-Pusp45::*sfgfp*	Em^r^; pSEUDO_10 derivative; vector for integration of *sfgfp* fusions into the *pseudo_10* locus of L. lactis in which Pusp45 drives sfGFP expression	20
pEPH01	Em^r^; pSEUDO_10-Pusp45::*sfgfp* derivative carrying the 5′ UTR of *thiT* upstream of the *sfgfp* coding sequence; used to construct strain EPH01	This work
pEPH02	Em^r^; pEPH01 derivative in which *sfgfp* was substituted for the coding sequence of the *acmA* gene; used to construct strain EPH02	This work

a RS*_thiT_*, *thiT* TPP riboswitch; UTR, untranslated region; *sfgfp*, superfolder green fluorescent protein gene; Em^r^, erythromycin resistance; Pusp45, constitutive promoter of the L. lactis *usp45* gene.

For microscopy experiments, plate reader assays, and flow cytometry, L. lactis was grown overnight in G-CDMPC without thiamine. The cells were resuspended (1:100) in fresh medium with different concentrations of thiamine hydrochloride (Sigma-Aldrich, Schnelldorf, Germany), incubated at 30°C, subsequently collected by centrifugation in the exponential growth phase at an optical density at 600 nm (OD_600_) of 0.4, and washed twice with a phosphate-buffered saline (PBS) solution prior to further use.

### General DNA techniques and transformation.

Purification of plasmid DNA was done according to the instructions of the manufacturer of the NucleoSpin plasmid kit and NucleoSpin gel. For the isolation of L. lactis chromosomal DNA, the cells of a culture grown overnight were spun down, resuspended in buffer A1 (10 mM Tris-HCl [pH 8.1], 10 mM EDTA, 50 mM NaCl) containing 10 mg/mL lysozyme (Roche Diagnostics, Mannheim, Germany), incubated for 60 min at 37°C, and further treated as described previously (48). DNA electrophoresis was performed in 1× TBE buffer (90 mM Tris-HCl [pH 8.3], 90 mM boric acid, 2 mM EDTA) in 1% 1× TBE agarose gels with 0.3 μg/mL ethidium bromide. Colony PCR was performed with *Taq* polymerase (Thermo Fisher Scientific Inc., Waltham, MA, USA), and the following cycle was repeated 33 times: denaturation at 98°C for 30 s, annealing at 50°C for 30 s, and elongation at 72°C for 120 s. Primers listed in Table 2 were purchased from Biolegio BV (Nijmegen, The Netherlands). Nucleotide sequencing was performed at Macrogen (Seoul, South Korea). E. coli transformation was done using the 42°C heat shock treatment protocol and chemically pretreated cells (49). Electrotransformation of L. lactis was performed using a Bio-Rad gene pulser (Bio-Rad Laboratories, Richmond, CA, USA) at 2.5 kV, 25 μF, and 200 Ω.

**TABLE 2:** Oligonucleotides used in this study

Primer	Sequence (5′–3′)
Fw. 0334	ATATGTTUGTAAAAATAAATATTTGCACAATGGGTCTATTG
Rv. 0334	AGCTCTTCUCCTTTTGACATAAAAAAAACTCCTTTTTGATGCGCACAAAAG
Fw. Bbpseudo	AGAAGAGCUGTTCACAGGTGTT
Rv. Bbpseudo	AAACATAUTATACTATTCCTACCCCACCTTA
Fw. acmA	ATGCCAGUATCACGTGTTAAAGTTAAAAATAGAC
Rv. acmA	AGTTTATUTTATTCGTAGATACTGACC
Fw. BB2pseudo	AATAAACUAGTCAAGGTCGGCAATTCTGCAG
Rv. BB2pseudo	ACTGGCAUAAAAAAAACTCCTTTTTGATGCG
Fw. pseudo	AAGCGGCCGCAGCATTCGTTGAACCTTTCATCATGCC
Rv. pseudo	GCTCTAGACAATTGCTCCCATGCTTGATTCC
Fw-RSthiT	GTAAAAATAAATATTTGCACAATGGG
Fw-BB3-SpeIx2	AGAAGAGCUGTTCACAGGTGTTACTAGTACTAGT
Rv-sfgfp	CTCATTATTACTTATAAAGCTCATCCATGCCG

### Construction of L. lactis strains.

To construct vector pEPH01 carrying RS*_thiT_*, the 5′ UTR of the *thiT* gene was amplified by PCR using uracil-containing primers Fw. 0334 and Rv. 0334 and 1 ng of L. lactis chromosomal DNA as the template. Plasmid pSEUDO_10-Pusp45::*sfgfp* was used to amplify the backbone using primers Fw. BBpseudo and Rv. BBpseudo. In both cases, DNA polymerase PfuX7 was used in the reaction. The insert and backbone PCR fragments were cleaned using the NucleoSpin gel and PCR cleanup kit (Macherey-Nagel GmbH, Düren, Germany). The PCR products were mixed (20 ng of the linearized plasmid and 200 ng of the insert fragment) with 1 U of User enzyme mixture (New England BioLabs, Ipswich, MA) in a total volume of 10 μL. The mixture was incubated for 30 min at 37°C, followed by 30 min at 25°C, and then used to transform competent cells of E. coli DH5α to pick up pEPH01. This plasmid, which cannot replicate in L. lactis, was introduced into L. lactis MG1363 via electroporation. Cells in which the plasmid had integrated into the *pseudo_10* genomic locus were obtained by erythromycin selection and subsequently selected by growth on selective SA medium plates containing 15 μg/mL of 5-fluoroorotic acid (5-FOA) (50). Finally, the insertion was confirmed by PCR and sequencing. For the construction of vector pEPH02, pEPH01 was used to amplify the backbone using the primers Fw. BB2pseudo and Rv. BB2pseudo, while the coding sequence of the gene *acmA* was amplified using the primers Fw. acmA and Rv. acmA. The strategy described above was utilized to obtain pEPH02. Fw-BB3-SpeIx2 was used for the mutation in EPH03. All plasmids and relevant chromosomal regions of the integration strains were verified by nucleotide sequencing.

### Spectrophotometry.

L. lactis strains MG1363 and EPH01 were grown and prepared as described above. For fluorescence intensity measurements, the cells were diluted 1:20 in G-CDMPC. When testing the effect of various concentrations of thiamine, this medium was supplemented with different amounts of thiamine. The growth (increase in the OD_600_) and fluorescence signal (excitation at 485 nm and emission at 535 nm) were recorded every 10 min in 0.2-mL cultures in 96-well microplates incubated for 22 h at 30°C using a Varioskan Lux microtiter plate reader (Thermo Fisher Scientific Inc., MA, USA). Fluorescence signals were corrected for the background value of the growth medium. sfGFP signals, in relative fluorescence units (RFU), were normalized by the corresponding OD_600_ measurements to obtain RFU/OD_600_ values.

### Flow cytometry.

L. lactis strains were grown overnight in G-CDMPC as described above and transferred to fresh G-CDMPC supplemented with various concentrations of thiamine. The cultures were incubated at 30°C, and samples were taken at exponential phase (OD_600_ of 0.4). The cells were washed twice in PBS and diluted 1:100. Afterward, a threshold of 200 was set for the forward-scatter (FSC) and side-scatter (SSC) parameters in the FACSCanto flow cytometer (GE Healthcare, San Jose, CA, USA) to remove all events not corresponding to cells. The sfGFP signal in all measured cells was recorded in 100,000 events. sfGFP signal measurements were obtained using a 488-nm argon laser. Data were collected using FACSDiva software 5.0.3 (BD Bioscience, CA, USA). FlowJo software version 7.6.1 (FlowJo LLC, Ashland, OR, USA) was used for data analysis.

### Microscopy.

L. lactis MG1363 strains Δ*acmA* and EPH02 were grown in G-CDMPC as described above. Exponentially growing cells (OD_600_ = 0.4) were washed twice with PBS, vortexed for 2 min, and transferred to a standard microscope slide carrying a solidified thin layer of PBS in 1.5% (wt/vol) high-resolution agarose (Sigma-Aldrich). A sample of 3 μL of the bacterial cell suspension was spotted onto the agar and covered with a standard microscope coverslip. Microscopic observations were performed using a DeltaVision microscope (Applied Precision, WA, USA). Images were acquired with a CoolSNAP HQ2 camera (Princeton Instruments, NJ, USA).

### Statistics.

Statistical analyses were performed using Prism 8 (GraphPad Software, La Jolla, CA, USA). Data are presented as means ± standard errors (*P < *0.05). For evaluations between two groups, Student’s *t* test was used. For comparisons of multiple groups, one-way analysis of variance (ANOVA) with Tukey’s test (*P < *0.05) was employed (51, 52). Three technical replicates were done in three independent experiments. The microscopy and flow cytometry pictures presented in this work are representative images from three independent replicate experiments.
